# Glycerine

**Published:** 1850-04

**Authors:** 


*Glycerine.*—By Mr. Amyot.—Glycerine has been of late undergoing the usual course of under-rating and over-rating to which every new remedy is invariably exposed; and as I think the "*contra*" side is now getting rather over-strong, I beg to declare myself among the "*pros*," for the good reason that I have used it much, and have found it most serviceable in a number of cases; so much so, that I believe I should be puzzled to find an efficient substitute for it. Such cases are the following:—

1. *Superficial burns*, whether the vesications are broken or not. Used with an equal quantity of water, and applied with a camel's hair brush. It gives great relief, and keeps the part moist for a long time. This is the most useful in burns of the face near the eyes, &c., or where dressings on lint, &c., cannot be well used.

2. In *sore nipples.* In theses it was first recommended, I believe, by Mr. Starlin, in the *Medical Times*, and I have never found any other application nearly so effectual. About one part to six or eight of water succeeds the best.

3. In the *excoriations* in the groins, nates, and behind the ears of infants.

4. In *porigo favosa and scutulata*, and other skin diseases, but especially in the first named (when the crusts have been removed by bread poultices. It should be used as a wash, diluted with eight or ten parts of water. I employ the pitch and sulphur ointment at the same time in these cases; but I do not find the latter so rapidly effectual *alone.*

My actual experience in the use of this remedy goes but little further; but I have no doubt it is very useful in chaps, &c., as Mr. McDougal has said, and, indeed, in any of the numerous class of cases where it is an object *to secure moisture of surface, and to protect from exposure to air.* At the least, I cannot consent, at the suggestion of another of your correspondents, to mount a crape hat-band, *as yet*, for glycerine.—*London Times.*

